# Exploring the interaction between extracellular matrix components of bone marrow and prostate cancer cells in a novel 3D model

**DOI:** 10.21203/rs.3.rs-10296973/v1

**Published:** 2026-09-02

**Authors:** Jianhui Yang, Peter Van Veldhuizen, Hani Awad, Hiroshi Miyamoto, Wojciech Wojciechowski, Kathleen Gillespie, Brian Marples, Omar Aljitawi

**Affiliations:** 1. Wilmot Cancer Institute, Department of Medicine, University of Rochester Medicine, Rochester, NY 14642; 2. Hematology/Oncology Division, University of Michigan Health-West, 5950 Metro Way Wyoming, MI 49519; 3. Center for Musculoskeletal Research, University of Rochester Medicine, Rochester, NY 14642; 4. Department of Pathology& Laboratory Medicine, University of Rochester Medicine, Rochester, NY 14642; 5. Flow Cytometry Resource, University of Rochester Medicine, Rochester, NY 14642; 6. Department of Radiation Oncology, University of Rochester Medicine, Rochester, NY 14642

**Keywords:** 3D culture, Prostate cancer, Bone marrow metastasis, Cancer stem cell

## Abstract

Our study aims to investigate the interaction between disseminated prostate cancer (PC) cells and the extracellular matrix (ECM) of the bone marrow microenvironment (BMM) using a 3Dimensional (3D) in vitro model, which biochemically resembles the stromal ECM part of the hematopoietic stem cell niche. Wharton’s Jelly (WJ) was processed and lyophilized to produce a decellularized WJ matrix (DWJM). PC cell lines DU145 and PC-3 were seeded on the DWJM to assess chemosensitivity, cell dormancy, and cancer stemness. CD133, a key marker of PC stem cells (PCSC), was used to evaluate the stemness of the PC cell lines by conventional and imaging flow cytometry. Our results demonstrated that both DU145 and PC-3 cells cultured on DWJM exhibited chemoresistance to Docetaxel and entered a dormant state. Additionally, a small population (~5%) of CD133(+) DU145 cells was detected after 7 days of culture on DWJM. These CD133(+) cells displayed the development of characteristics of epithelial-to-mesenchymal transition. Moreover, limited dilution assay in vivo revealed that DWJM-induced CD133(+) DU145 cells were significantly more tumorigenic than parental DU145 cells. Our findings suggest that PCSCs may be induced in DU145 cells but not in PC-3 cells when PC cells cultured on DWJM. In conclusion, we have developed a 3D *in vitro* model that effectively mimics the interaction between disseminated PC cells and the ECM of BMM, which may inform the development of therapies targeting metastatic cells in BMM.

Prostate cancer (PC) is the most commonly diagnosed malignancy and the second leading cause of cancer-related death in men in the United States^1^. Bone is the most frequent site of metastasis from PC. A study of 1,589 autopsy cases who died with PC found that 90% had bone involvement^2^. Bone metastasis is a major contributor to PC-related mortality, as disseminated PC cells within the bone marrow microenvironment (BMM) exhibit significant resistance to current treatments. However, the precise mechanisms underlying this treatment resistance remain poorly understood.

The dormant state of disseminated tumor cells (DTCs) in the BMM is a leading theory to explain treatment resistance in PC as well as other solid cancers^3–5^. Dormancy is not simply a survival mechanism for DTCs in unfriendly or hostile tumor environments, it also represents a phenotype associated with increased stemness. In some cases, the dormant DTCs may further their development into quiescent cancer stem cells (CSCs)^6^. Moreover, DTCs with enhanced stem cell-like properties in the BMM can serve as the source of secondary cancer spread to distant organs, including the brain, lungs, and liver^7^.

The hematopoietic stem cell (HSC) niche, where HSCs maintain their quiescent state, may represent the initial site of PC cell metastasis to the BMM^8^. HSC niche is also likely the primary location where DTCs adopt a dormant state, interact with quiescence-associated signaling, and increase their stemness. The drug resistance observed in metastatic PC within the BMM may be driven by complex interactions between DTCs and the HSC niche, which consists of both cellular and non-cellular components. While much has been published on the role of cellular components of the HSC niche such as mesenchymal stromal cells, osteoblasts, and adipocytes^9–11^, the contributions of the non-cellular components remain poorly understood. The extracellular matrix (ECM), a key non-cellular component of the HSC niche, plays a critical role in the tumor microenvironment (TME)^12,13^, adult stem cell niches^14,15^, and CSC niches^16–18^. Thus, in addition to cell-cell interactions, the cell-ECM interactions in the BMM should also be investigated when evaluating the complex molecular and cellular dynamics between DTCs and the whole HSC niche.

Three-dimensional (3D) culture systems can replicate the *in vivo* environment, bridging the gap between 2D cultures and animal models. In particular, 3D *in vitro* models that emulate HSC components allow for a more focused exploration of the interactions between DTCs and non-cellular elements of the HSC niche^19,20^. Compared to animal models, 3D culture offers two main advantages for studying the BMM. First, animal models may not accurately reflect human BMM pathology and disease progression due to species-dependent differences. Second, isolating and studying the ECM of BMM in mice is technically challenging, whereas 3D biomatrix cultures allow for a more straightforward exploration of cell-ECM interactions. Therefore, the 3D biomatrix model can complement animal studies and serves as an effective tool for investigating cell/ECM interactions in the BMM.

The ECM is not only a simple structural and mechanical framework but also a potent modulator of some key cellular functions. However, there is a lack of *in vitro* models that adequately mimic features of the ECM of HSC niche in a high throughput manner. Wharton’s Jelly (WJ) is the ECM part of the umbilical cord, with biochemical components resembling the counterpart of the BMM-derived stromal ECM. After the decellularization of WJ, we developed a biomimetic ECM model of BMM which we refer to as Decellularized WJ matrix (DWJM). This DWJM is engineered as a porous scaffold which is suitable for cells to reside inside, and biochemically it contains an abundance of the major HSC niche proteins^21^. Our previous work demonstrated that DWJM scaffold can effectively maintain the viability, colony-forming capability, and expansion of umbilical cord blood (UCB) CD34(+) cells by providing major HSC niche components^22^. Therefore, the 3D DWJM model can mimic the ECM of the HSC niche and cell/ECM interaction when PC cells metastasize to BMM. Data collected from this study provide some initial insights into dormancy, chemoresistance, and cancer stem cell-like properties in prostate cancer cells within the BMM-like DWJM.

## Results

### 1. Features of DWJM and observation of PC cell growth in the presence of DWJM

WJ is the gelatinous substance surrounding umbilical vessels (Supplementary Fig. S1) and was harvested by stripping the umbilical blood vessels and membranes (Fig. 1A). After decellularization and lyophilization, the WJ was processed into porous DWJM wafers (Fig. 1B) and stored at −20°C until use. In our previous work^21^, we analyzed DWJM by mass spectrometry, revealing key components that mimic the ECM of the BMM as summarized in Fig. 1C.

In our previous work, electron microscopy revealed that DWJM wafers have numerous pores that support cell residence within the matrix^21^. After seeding PC-3 or DU145 cells stained with CellTracker Green, fluorescence microscopy imaging showed that the majority of PC cells were either embedded within or adhering on the surface of the DWJM. The growth patterns of PC cells within the microporous scaffold of DWJM formed irregular cell clusters (Fig. 1D). After dissociation with collagenase and re-culturing in a flask or dish, both cell lines self-assembled into spheroids, with DU145 cells showing a more pronounced spheroid formation (Supplementary Fig. S2).

### 2. Evaluation of chemosensitivity, dormancy and EMT phenotype of PC cells in DWJM 3D culture

After culturing DU145 or PC-3 cells in the presence of DWJM for 3 days, cells were dissociated with collagenase and then plated onto tissue culture polystyrene (TCP) for 2D culture. Following overnight incubation, cells were treated with Docetaxel at concentrations of 0.1 or 1 nM for 48 hours. MTS cell viability assays revealed that DWJM-cultured DU145 or PC-3 cells showed significant resistance to Docetaxel (Fig. 2A). Given that cancer cell dormancy is one of main contributors to drug resistance, we next investigated whether PC cells dissociated from DWJM exhibited changes in cell proliferation and Ki-67 expression. MTS proliferation assays showed that both DU145 and PC-3 cells dissociated from DWJM exhibited slower growth rate over 6 days compared to control cells not grown in DWJM (Fig. 2B). Additionally, flow cytometry analysis revealed that DU145 cells, but not PC-3 cells, had lower expression of Ki-67 (Fig. 2C).

Epithelial-mesenchymal transition (EMT) facilitates metastasis of primary tumor cells to distant organs and contributes to treatment resistance observed in metastatic cancer cells within the BMM. Both DU145 and PC-3 are metastatic PC cell lines and our preliminary data showed that these two PC cell lines exhibited relative low expression of E-cadherin and high expression of Vimentin (data not shown), indicating the preexisting EMT characteristics in these cell lines. Among other EMT markers, EpCAM is considered to reflect a later stage in the EMT process^23^. As shown in Supplementary Fig. S3, both DU145 and PC-3 cells demonstrated high EpCAM expression, suggesting that these two cell lines are perhaps in an intermediate stage of the transition to mesenchymal phenotype. We then further checked the EpCAM expression for possible EMT progress after cell culture in the presence of DWJM. Notably, EpCAM expression was significantly decreased in DU145 but not PC-3 cells after 3 days of culture with DWJM (Fig. 2D).

### 3. Evaluation of CSC characteristics after DWJM culture in vitro

In addition to dormancy, DTCs within the BMM may also exhibit characteristics resembling CSCs^24^. In this study, we used our biomimetic ECM model to specifically investigate whether interactions between PC cells and ECM would influence the stemness of PC cells in the BMM. One of the main challenges in distinguishing prostate CSCs (PCSCs) from dormant DTCs is choosing appropriate markers of CSCs for PC cells, because there is no consensus on definitive PCSC markers, likely due to the heterogeneity and plasticity of cancer cells. Current potential markers for PCSCs include CD133, CD44, CD338 (ABCG2), CD49b (Integrin α-2), and ALDH1A1 (a key member of the ALDH1 family), or combinations of these markers. Among these tentative PCSC markers, CD133 alone or in combination with other markers is the most widely used^25,26^.

As shown in Fig. 3A, flow cytometry analysis revealed that both DU145 and PC-3 cells expressed moderate to high levels of most of the tentative PCSC markers, with the exception of CD133. Furthermore, Western blot and reverse transcription-polymerase chain reaction (RT-PCR) analysis confirmed that CD133 expression in both DU145 and PC-3 cells was nearly undetectable (Fig. 2B). The absence of CD133 expression in these PC cells aligns with the core definition of CSCs, which defined that an ideal CSC marker should be minimally expressed in the bulk tumor or tumor-derived cell lines. What is more, CD133 is possibly a master marker of progenitor cells at the apex of the CSC hierarchy, as numerous studies indicate that CD133(+) cells have enhanced self-renewal capacity, tumorigenic potential, and resistance to therapy. Therefore, we selected CD133 alone as the primary PCSC marker for DU145 and PC-3 cells, consistent with several other studies that have used CD133 alone to identify PCSCs^27,28^. After culturing PC cells in the presence of DWJM for 7 days, flow cytometry analysis showed that CD133 expression in DU145 cells increased to 4.90 ± 1.57% of the total cell population, compared to 0.17 ± 0.11% in untreated cells (Fig. 3C). Imaging flow cytometry further confirmed that the staining of CD133 was on the cell membrane of viable cells (Fig. 3D). Interestingly, PC-3 cells did not show a significant increase in CD133 expression under the same experimental condition as shown by flow cytometry analysis (Fig. 3C).

### 4. Evaluation of CSC characteristics of sorted CD133(+) cells induced by DWJM in vitro

The previous work has shown that cell-ECM interactions in this DWJM model induced a small population of CD133(+) DU145 cells, which was subsequently sorted for further analysis. Flow cytometry revealed significantly reduction of EpCAM expression on sorted CD133(+) cells compared to parental cells (Fig. 4A). Next, we assessed the growth and survival of these sorted CD133(+) cells. Unlike traditional ways of assessing cell growth (such as MTS), which usually require large number of cells and are limited in a short term, colony forming assay (CFA) can be used to observe cell proliferation of a small number of cells particularly stem cells or CSCs in a longer term. In this study, a two-week CFA demonstrated that CD133(+) DU145 cells formed significantly fewer colonies than the parental cells, with the colonies being much smaller in size (Fig. 4B). These findings suggest that the sorted CD133(+) cell population is in a quiescent state. Microscopely, the colonies formed from CD133(+) cells exhibited an increased nuclear-to-cytoplasmic ratio and more elongated, spindle-shaped morphology, which is consistent with the shape and morphology of mesenchymal cells (Fig. 4C). Moreover, the sorted CD133(+) cells growing in cell chamber were assessed for key EMT markers using an EMT Immunohistochemistry (IHC) kit. Compared to parental cells, CD133(+) cells showed reduced E-cadherin expression and increased vimentin expression, but no significant elevation of Snail expression was observed as expected (Fig. 4D).

### 5. Evaluation of Tumorigenic ability of DWJM enriched CD133(+) cells in vivo

A preliminary and a small scale Limited Dilution Assay (LDA) was conducted to determine the proper cell number range required for tumor formation. In our preliminary experiment, it was found that the minimal numbers of parental cells and CD133(+) cells to form tumors in nude mice were 10^6^ and 10^3^, respectively (see Supplementary Fig.S4). Nevertheless, CD133(+) cells took significantly longer time to form tumors compared to parental cells (22 weeks *vs* 12 weeks), aligning with the dormant state of CSCs. Subsequently, 20 nude mice were divided into 4 groups (5 mice/group) and subcutaneously injected with either DWJM-induced CD133(+) cells or control parental cells at doses of 1 × 10^4^ or 1 × 10^3^ per injection. Approximately five months post-inoculation, tumor formation was observed in 3 out of 5 mice injected with 1 × 10^4^ CD133(+) cells and in 1 out of 5 mice injected with 1 × 10^3^ CD133(+) cells. In contrast, the same numbers of parental cells did not result in tumor formation (Fig. 4A). Analysis of the LDA data using ELDA software revealed that the tumor formation rate of CD133(+) cells was statistically higher than that of parental DU145 cells.

Other than the role in EMT, EpCAM can also be used as a marker to detect bone marrow (BM) metastasis originated from epithelial derived cancers including PC. EpCAM expression is not typically found on most hematopoietic cells, but it expresses at low level of hematopoietic stem and progenitor cells (HSPCs) which is a rare population of all BM cells. In this study, the EpCAM expression level of BM cells (BMCs) isolated from mice without tumor formed is around 0.17±0.12%. In contrast, 2 out of 4 BM samples from mice bearing tumor formed from CD133(+) cells have elevated EpCAM expression level to 2.36 ± 0.83%, possibly indicating more malignant and metastatic characteristic of CD133(+) cells metastasizing into the BMM.

## Discussion

In this study, we employed our DWJM-based model to mimic the ECM components of HSC niche *in vitro*, aiming to observe phenotypic changes in PC cells following cell-ECM interactions within BMM. Our main findings demonstrated that DU145 cells, but not PC-3 cells, in this DWJM model acquired chemoresistance, which can be contributed to a dormant state and progression of partial EMT phenotype of PC cells, as well as the induction of putative PCSCs.

The influence of the BMM on hematopoiesis or DTCs has been primarily studied in the context of cell-to-cell interactions, with cell/ECM interactions often overlooked^29^. This oversight is partly due to the technical challenges associated with exclusively exploring the entire ECM of BM components in animal models. This project is motivated by the need to recapitulate the ECM of BMM by an ECM-based 3D-biomatrix *in vitro*, to better understand the interaction of cells/ECM. Notably, this novel 3D biomatrix mimics more closely the natural ECM of human cells and may offer advantages over alternatives such as Matrigel, which resembles basement membrane and is derived from Engelbreth Holm Swarm (EHS) mouse sarcoma cells, poorly differentiated chondrosarcoma cells^30^. More importantly, our previous studies have shown that DWJM can expand UCB CD34(+) cells while preserving their differentiation potential^22^. This is largely due to the chemical similarities between DWJM and the BMM^21^ as summarized in Fig.1C, as well as the porous structure of the scaffold where cells reside and adhere tightly. Given these properties, DWJM was proved to be well-suited for culturing HSCs *ex vivo*^22^ and could potentially be applied to investigate cell-ECM interactions after PC cells metastasize to the BM HSC niche.

Docetaxel provides significant survival benefits for patients with castration-resistant PC and is commonly used as a first-line chemotherapy for those with metastatic PC. Both the DU145 and PC-3 cell lines, which were derived from metastatic prostate cancers, respond well to Docetaxel in 2D culture. In the decellularized ECM 3D model, PC cells sensed surrounding ECM proteins and underwent substantial change after culturing with DWJM. The MTS viability assay showed that both dissociated PC cell lines manifest significant resistance to Docetaxel. Moreover, upon returning to 2D culture, both PC cell lines retained reduced proliferation after being cultured with DWJM. Additionally, flow cytometry analysis further showed a decreased expression of Ki-67 in DU145 cells, indicating that these slow growing cells may have entered a quiescent state. Taken together, it is reasonable to conclude that the PC cell-ECM interaction in the DWJM 3D culture model may drive PC cells to chemoresistance at least partly due to the induced dormant mode, which aligns with recent studies suggesting that ECM components in TME can trigger proliferation arrest and regulate dormancy of cancer cells^24,31,32^. Furthermore, accumulating evidence also highlights ECM components play a significant role in drug resistance via regulating EMT^33,34^. We further hypothesize that the progression of EMT, in addition to a dormant state, may also contribute to chemoresistance of PC cells growing on DWJM. In support of this, flow cytometry was performed to observe the expression of EpCAM, a key marker involved in the progress of EMT. It was found that the expression of EpCAM was decreased in DU145 cells but not PC-3 cells after culturing within DWJM for 3 days, suggesting the development of EMT phenotype in DU145 cells.

As mentioned earlier, dormancy theory remains the predominant explanation for the treatment resistance of DTCs in the BMM. However, it remains unclear whether a small subset of dormant DTCs have been further converted into CSCs through cell/ECM interactions within BMM. While there is substantial overlap between dormant cancer cell populations and CSCs^35^, CSCs are a distinct population characterized by specific surface markers, enhanced tumorigenicity *in vivo*, and self-renewal properties, etc. Recently, a few reports have provided preliminary evidence suggesting that DTCs in the BMM may acquire increased stemness following cell-cell interaction^7,24,31^. These studies also indicate that the BM niche may be a critical site where DTC reprogrammed to a CSC phenotype^31^. In this DWJM model, we specifically investigate whether cell-ECM interactions influence the stemness phenotype of PC.

By definition, CSCs are supposed to be capable of initiating tumor growth with very a few cell numbers. CSCs are typically identified by their characteristic expression of certain molecular markers, which can be used to isolate these cells for further assessing their stemness through animal transplantation assays such as LDA. Based on our preliminary data of LDA (Supplementary Fig. S4), although highly expressed most tentative CSC markers except CD133, the DU145 cell line had limited tumorgenicity because it required at least 10^6^ cells to form tumors in nude mice. In this study, CD133 alone was selected as the tentative PCSC marker for the following reasons: (1) Most of highly expressed markers as shown in Fig.3A, such as CD44, may not be PCSC specific for these two PC cell lines. Because if CD44 were truly a PCSC marker, DU145 cells should form tumors at much lower cell numbers; (2) CD133(+) cancer cells have been shown to form tumors at cell numbers as low as 10 or 100^32,33^; and (3) The nearly undetectable level of CD133 in parental PC cells (Fig. 3A) suggests its rarity in tumor bulk, supporting our hypothesis that CD133 is more likely to be a stronger PCSC marker, or closer to the apex of the PCSC hierarchy.

After selecting CD133 as the tentative PCSC marker for this study, we observed the expression of CD133 in DU145 cells after culturing with DWJM for 7 days. We found that CD133 expression was induced in approximately 5% of the DU145 cells. Besides, imaging flow cytometry further confirmed the typical membrane staining of CD133 protein on viable cells. However, it remains unclear why PC-3 cells were not induced to express CD133 in this model. It was reported that DU145 cells showed less pronounced EMT characteristics compared to PC-3. Interestingly, full EMT tends to drive cells towards a fully mesenchymal state, which may not always align with the characteristics of CSCs^35^. Besides, PC-3 cells might be more reflective of rare neuroendocrine PC cells, while most PCs are adenocarcinoma. DU145 cells might be advantageous in PCSC research in terms of the representation of adenocarcinoma.

EpCAM is a transmembrane glycoprotein commonly used as a marker for epithelial cells. It is a key epithelial marker whose expression decreases during EMT, as cells transition from an epithelial to a mesenchymal state. After cell sorting, the CD133(+) DU145 cells exhibited a significant decreased level of EpCAM, which was indicative of transition toward a mesenchymal phenotype. Additionally, a 2-week CFA revealed that CD133(+) cells forms fewer and smaller colonies compared to parental DU145 cells. This finding can be explained by the dormant state of CD133(+) cells, which is consistent with another important feature of CSCs, known to be either quiescent or to proliferate much more slowly than non-CSCs. Under bright-field microscopy, the cell morphology of CD133(+) colonies appeared elongated, which aligns with a mesenchymal phenotype. Besides, the development of EMT were further characterized by staining with antibodies from an EMT IHC kit. The IHC result showed significantly decreased E-cadherin and markedly increased vimentin, but the snail expression was not significantly elevated as expected. This suggests that the CD133(+) cells are undergoing EMT but is still hybrid phenotype, which is generally considered to be closer to the CSC state than a full EMT^36^.

It is widely accepted that the gold standard for evaluating CSC characteristics is LDA *in vivo*^29^. CSC theory posits that only a subpopulation of cancer cells within a tumor is capable of initiating and sustaining tumor growth, while the majority of the population consists of non-CSCs that lack tumor-initiating potential. In our LDA study, DWJM induced CD133(+) DU145 cells demonstrated the ability to initiate tumor growth at a lower cell number at 10^3^-10^4^, whereas parental DU145 cells at same number failed to form tumors. What is more, 2 out of 4 BM samples from mice injected with CD133(+) cells were detected to have elevated EpCAM expression, possibly suggesting a more metastatic profile of CD133(+) cells and BM metastasis. Further analysis of BM samples from more mice with tumors formed from injected CD133(+) cells is necessary to draw a clearer conclusion.

In summary, the key finding of this study is that DWJM induced approximately 5% CD133(+) DU145 cells following cell-ECM interactions, supporting the hypothesis that the ECM of BMM can promote the conversion of cancer cells into CSCs. However, the specific components of the ECM that activate the pathways responsible for CD133 re-expression still require further investigation. Future research will focus on two potential pathways. One pathway is CD44-HA interaction as the majority of DU145 cells express CD44 (Fig. 3) and one of the primary ligands of CD44^37^ is HA, which is highly expressed at DWJM (see Supplementary Fig. S5). The binding of CD44 to HA could activate various intracellular signaling pathways, some of which might be involved in reprogramming PC cells to a CSC-like phenotype. The other pathway is related to collagen, which influences various aspects of tumor progression when binding to its receptor integrin^38^. Collagen is abundant in DWJM as detected in our previous study^21^. As shown in Fig.3, DU145 cells express moderate level of integrins, which might bind to collagen and trigger some important cellular and molecular events related to the induction of CSC phenotypes. The ultimate goal of cell/ECM research in BMM is to find a way to block its interaction, which may pave a new road to treat or prevent PC bone metastasis.

## Materials and Methods

### 1. Chemicals and reagents

Most chemicals were dissolved in dimethyl sulfoxide (DMSO) to create stock solutions at 1000 times the working concentration. The final DMSO concentration in the working solution was 0.1% (v/v) or lower, which did not exhibit any noticeable inhibitory effects on cultured cells.

### 2. WJ Decellularization

Decellularization of WJ was performed as described in our previous publications with minor modifications^21,39^. Discarded and de-identified umbilical cords were collected from the Department of Obstetrics at the University of Rochester Medicine using an exempt protocol approved by the Research Subjects Review Board (RSRB No. 00065499), which is the Institutional Review Board (IRB) of the University of Rochester Medicine. This study was conducted in accordance with the guidelines of the Declaration of Helsinki. In brief, the fresh human umbilical cords were washed multiple times with PBS to remove blood components, then cut into 5–6 cm segments. Vascular structures and surrounding membranes were carefully dissected and removed. Then WJ was decellularized by exposing to hypertonic and hypotonic solutions, a non-ionic detergent (Triton-X 100), an anionic detergent (sodium lauryl succinate), and enzymatic digestion (recombinant endonuclease). The decellularized WJ was rinsed in PBS to neutralize the pH, then placed in a 3 mm thick mold for lyophilization. After lyophilization, the scaffolds were sterilized by UV irradiation for 2 hours and stored at −20°C.

### 3. Cell culture and cell seeding on DWJM for 3D culture

Human PC cell lines DU145 and PC-3 were obtained from the American Type Culture Collection (ATCC, catalog # CRL-1435 and HTB-81) and maintained in F-12K media and RPMI-1640 media, respectively. Both mediums were supplemented with 10% fetal bovine serum (Gibco, Cat#56707–01) and 1% penicillin/streptomycin solution (Thermo Scientific, Cat#15140122). The preparation of 3D cultures followed protocols from our previous publication^40^ and similar method used by other labs^41,42^. DWJM was cut into approximately 25-mm diameter circular pieces using sterile scissors and placed in 12-well low-attachment tissue culture plates (Thermo Scientific, Cat# 174931). A 200-μl volume containing 4×10^5^ cells was seeded dropwise on DWJM, then the plates were incubate for 2 hours so cells can infiltrate and incorporate into DWJM. Each 12-well plate was then filled with 2 mL of media, which was replaced every 2 days.

### 4. Fluorescence imaging of DWJM culture

The lyophilized CellTracker Green (Thermo Fisher, Cat# 7025) was dissolved in DMSO to a 10 mM stock solution. This stock was then diluted to a final working concentration of 10 μM (1:1000 dilution) in serum-free medium. PC cells were stained with the dye for 30 minutes in a cell culture incubator, then washed with DPBS. The stained cells were resuspended in culture media at a concentration of 10^5^cells/100 μL and seeded onto DWJM.

### 5. Conventional and imaging flow cytometry analysis

A total of 10^6^ cells were washed and resuspended in fluorescence-activated cell sorting (FACS) buffer (2% FBS in PBS). Viability staining was performed by incubation with 1 μL Ghost Dye^™^ Red 780 Fixable Viability Dye (Cell Signaling Technology, Cat# 18452) in 1 mL FACS buffer for 30 minutes on ice. Following a wash, cells were stained for CD133 using 1 μL human CD133-Alexa Fluor 488 antibody (Cell Signaling Technology, Cat# 38725S) in 100 μL FACS buffer for 1 hour on ice in the dark. The samples were analyzed using an LSR II flow cytometer with FACSDiva software (BD Biosciences, and CD133 expression on viable cells was quantified using FlowJo Software (Tree Star). CD133 (+) cells were identified and quantified by excluding debris, doublets and dead cells, followed by compared to untreated parental cells. Imaging flow cytometry was performed in parallel on an ImageStream X Mark II (Cytek Biosciences), and data were analyzed with IDEAS software. CD133 cells were specifically identified by membrane localization of positive CD133 signal and negative viability staining.

For intracellular staining with a Phycoerythrin conjugated anti Ki-67 antibody (BioLegend, Cat# 350503), cells were fixed in 2 mL of cold 70% ethanol dropwise while vortexing to ensure proper fixation. The fixed cells were incubated at −20°C for 1 hour. After fixation, cells were incubated with 5 μL of Ki-67 antibody for 30 minutes at room temperature in the dark before analysis.

### 6. Cell Sorting

Single-cell suspensions were stained and resuspended at a concentration of 1×10^7^ cells/mL in sterile PBS. CD133 (+) cells were isolated by FACS using a FACSAria II (BD Biosciences. Cells were sorted at a sheath pressure of 45 psi with an 85 μm nozzle. Sort gates were set based on CD133-Alexa Fluor 488 (+) and Ghost Dye^™^ Red 780 Fixable Viability Dye (−) on the dot plot to isolate viable CD133(+) cells.

### 7. Chemosensitivity assay by MTS

The chemosensitivity assay was performed using the CellTiter 96^®^ AQueous MTS Cell Proliferation Kit (Promega, Cat# G5421), following the manufacturer’s protocol. DU145 and PC-3 cells, cultured with DWJM for 3 days, were seeded in a 24-well plate at 10,000 cells/well and incubated overnight. The cells were then treated with Docetaxel at concentrations of 0.1, 1, and 10 nM for 24 and 48 hours. After treatment, MTS reagent was added to the cells, and the absorbance of the developed color was measured at 490 nm using a BioTek Synergy^™^ ǁ Multi-Detection Microplate Reader. Cell viability was calculated as the percentage of viable cells relative to untreated controls.

### 8. Western blotting

Cell and tissue lysates were prepared using lysis buffer (Pierce), and protein was extracted from cells cultured on 6-well plates. Equal amounts of protein were loaded onto a 10% SDS-PAGE gel and transferred to a nitrocellulose membrane using a tank blotting system (Bio-Rad). Membranes were incubated overnight with CD133 (D2V8Q) XP^®^ Rabbit mAb (Cell Signaling Technology, Cat# 64326), followed by incubation with an anti-rabbit HRP-linked secondary antibody (Cell Signaling Technology, Cat# 7074). GAPDH (D16H11) XP^®^ Rabbit mAb (Cell Signaling Technology, Cat# 5174) was used as a loading control.

### 9. Semi-quantitative RT-PCR

Total RNA was extracted from all cell lines using the RNeasy Mini Kit (Qiagen, Cat# 74104), and cDNA was synthesized using the GoScript^™^ Reverse Transcriptase cDNA Synthesis Kit (Promega, Cat# A5000). PCR was performed by T100 Thermal Cycler (Bio-Rad) using primers as follows: CD133: 5’-TAC CTG GTG ATT TGC CAC AA-3’, 5’- CTG GGG CTG CTG TTT ATT A-3’; β-actin: 5’- CTT CCA GCC TTC CTT CCT G-3’, 5’- TTG GCG TAC AGG TCT TTG C–3’. PCR products were analyzed on a 1.5% agarose gel. PCR cycling conditions were optimized to fall within the linear amplification phase for comparative quantification.

### 10. CFA

CD133(+) cells and parental control cells were seeded in 6-well plates at densities of 100, 200, or 400 cells per well, with the medium replaced every 3–4 days. Colonies were fixed in ice-cold methanol for 10 minutes on ice, then stained with 0.05% crystal violet (Sigma, Cat# 46364) for 15 minutes at room temperature. After staining, colonies were rinsed with PBS and allowed to air dry overnight. Colony formation was assessed by visual inspection.

### 11. Evaluation of EMT by IHC

Cells were grown on an 4-well Nunc^™^ Lab-Tek^™^ chambered slide (Cat#177437, Thermo Scientific), fixed with 100% paraformaldehyde for 15 minutes at 37 °C, followed by blocking in 0,3% Triton X-100, 1% BSA, and 10% normal donkey serum in PBS for 1 hour. Evaluation of the differentiation status of the cells was performed by staining with antibodies against three main EMT markers NL637-conjugated goat anti-human E-cadherin, NL557-conjugated goat anti-human Snail, and NL493-conjugated goat anti-human vimentin using an EMT 3-Color Immunocytochemistry kit (R&D systems, Cat# SC026) according to the instructions of the manufacturer. Cells were mounted using SlowFade^™^ Gold Antifade Mountant (Invitrogen, Cat# S36940) before images were generated using Stellaris 5 inverted confocal microscope (Leica).

### 12. Limited Dilution Assay in vivo

The tumor-initiating capacity of DWJM-induced CD133(+) DU145 cells and parental DU145 cells was evaluated through subcutaneous inoculation of varying cell numbers into the flanks of nude mice. All NU/J athymic nude mice (age 6–8 weeks, male) were purchased from Jackson Lab (Strain#: 002019). The mice were housed under controlled light conditions and provided water *ad libitum*. The mouse experiment was approved by the University Committee on Animal Resources- University of Rochester (approval number: UCAR 2023–004) and performed in compliance with the NIH and the ARRIVE guidelines. A preliminary experiment was used to determine the optimal cell number for injection. Five mice per group were administered with either parental DU145 cells or DWJM-induced CD133(+) cells at doses of 10^4^ or 10^3^ cells per group. Cells were resuspended in 50 μl PBS mixed with 50 μl Matrigel^®^ Growth Factor Reduced Basement Membrane Matrix (Corning, Cat# 356230) and injected into the mice subcutaneously. The mice are then monitored for tumor burden for four months and the final readout is the tumor incidence per cohort. When ulceration occurred at the site of tumor growth or tumor volumes exceeded 1000 mm^3^, mice were humanely euthanized by CO_2_ asphyxiation (displacement rate: about 30% chamber volume/min) followed by cervical dislocation. The frequency of tumor-initiating cells was analyzed using Extreme Limiting Dilution Analysis (ELDA) software (https://bioinf.wehi.edu.au/software/elda/)^43–45^.

### 13. BMC isolation.

Whole BMCs were harvested from the femurs and tibiae of mice with CD133(+) tumors. The BMCs were isolated by flushing the bones with PBS containing 2% FBS using 25G needles. Red blood cells were lysed using Gibco ACK Lysing Buffer (Invitrogen, Cat# A1049201)

### 14. Statistical analysis

Data are expressed as the mean ± standard deviation. All statistical analyses were performed using Excel except ELDA for LDA data. Significance was determined using a two-tailed Student’s t-test, and *P* < 0.05 was considered statistically significant.

## Supplementary Files

This is a list of supplementary files associated with this preprint. Click to download.

- Supplementaryinformation.docx
- Supplementaryinfo20260722.pdf
- UCARapprovalnotice20260811.pdf

## Figures and Tables

**Figure 1.**
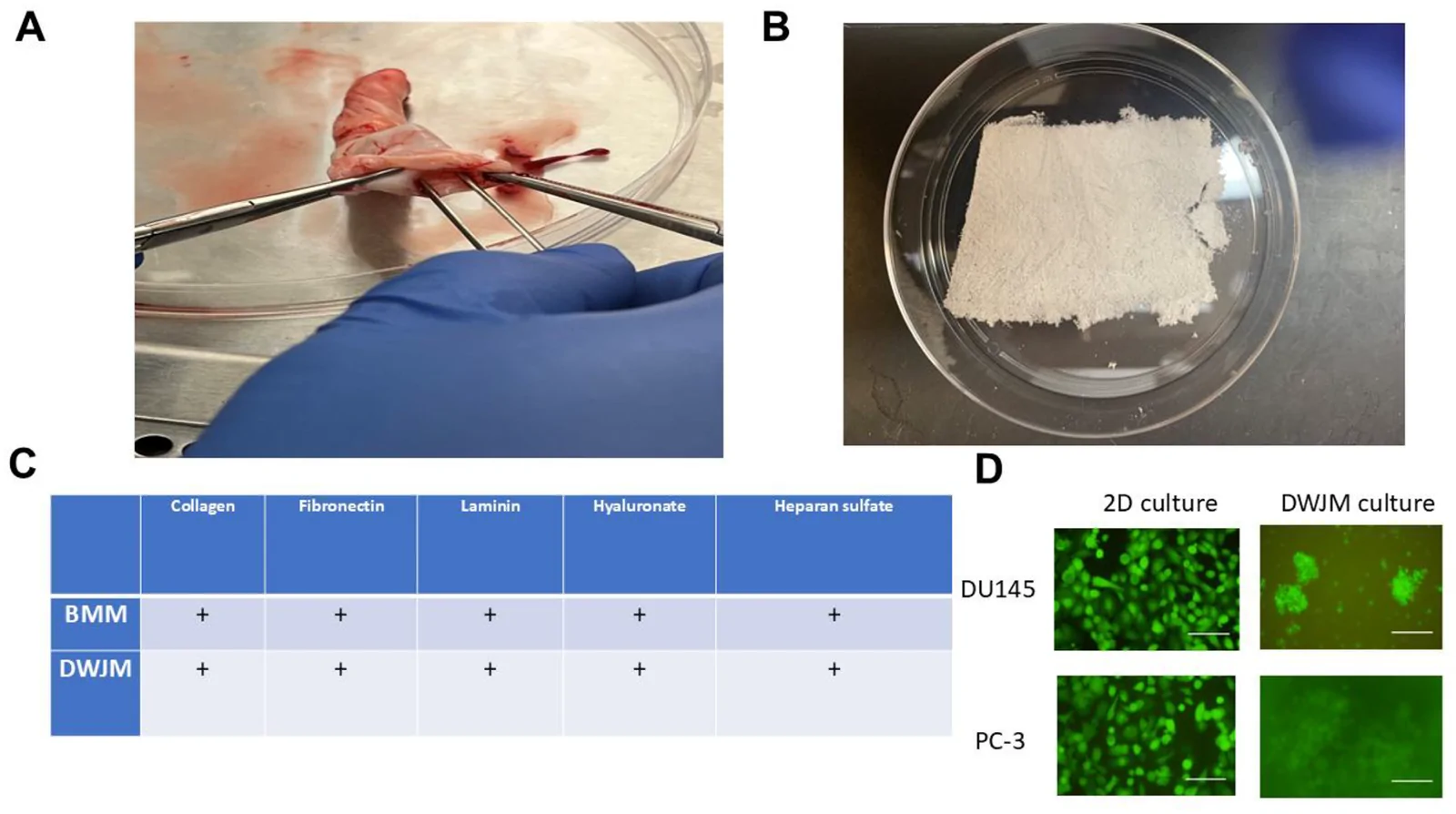
Features of DWJM and observation of PC cell growth in the presence of DWJM. **(A)** Umbilical blood vessels and sub amniotic envelopes stripped to isolate WJ. **(B)** Appearance of Decellularized WJ Wafer after lyophilization. **(C)** Lyophilized DWJM contains many main ingredients of BMM^21^. **(D)** Fluorescence imaging showed growth patterns of PC cells in the microporous scaffold of DWJM when compared with 2D culture. Scale bar represents 100 μm.

**Figure 2.**
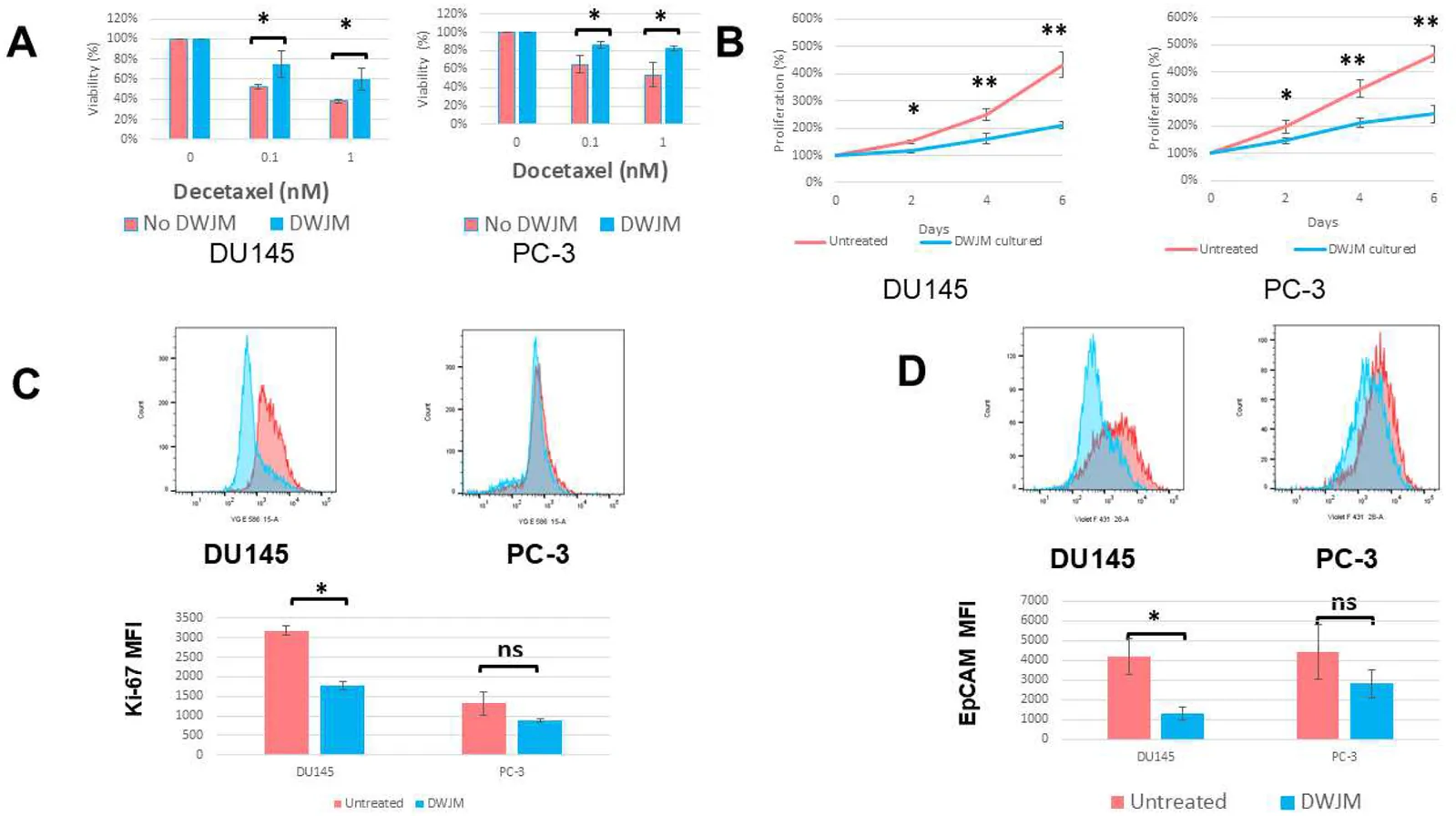
DWJM induces drug resistance, dormant state and partial EMT of PC cells. **(A)** The sensitivity of DWJM-cultured DU145 and PC-3 cells to Docetaxel at the concentrations of 0.1 or 1 nM for 48 hours. **(B)** Proliferation of DWJM-cultured DU145 and PC-3 cells over a time period of 6 days. The Ki-67 **(C)** and EpCAM **(D)** expression levels in DU145 and PC-3 cells after 3 days culturing with DWJM. Orange and blue lines in FCM histogram represent control and DWJM cultured cells respectively. The mean fluorescence intensity (MFI) is used to quantify the level of protein expression. Data are expressed as the mean ± standard deviation from 3 independent experiments. *p<0.05, **p<0.01, ns indicates non-significance.

**Figure 3:**
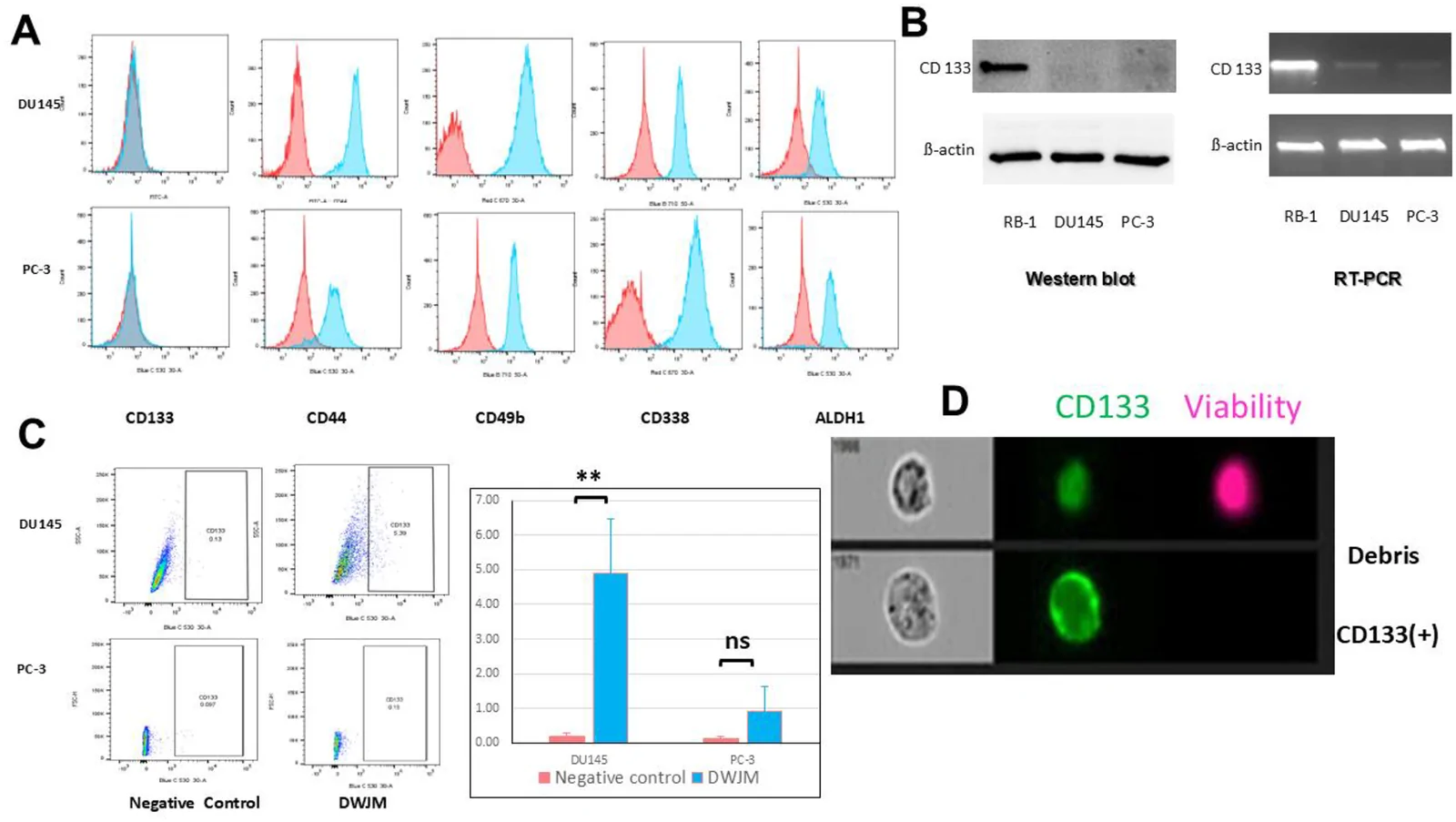
DJWM induces CD133 expression in DU145 cells. **(A)** The basal level of tentative PCSC markers in PC-3 and DU145 cells. Orange and blue lines in FCM histogram represent unstained and stained with antibody. **(B)** Western blot (left) and RT-PCR (right) analysis of CD 133 in DU145 and PC-3 lines. WERI-Rb-1(RB-1) cell was used as positive control in this experiment. **(C)** After culturing DU145 or PC-3 cells with DWJM for 7 days, CD133 expression was quantified after gating out debris, doublet, and dead cells(left). We performed 6-independent experiments, and data were expressed as the mean ± standard deviation (right). ns indicates non-significance; *p<0.05; **p<0.01. **(D)** Imaging flow cytometry analysis of cell morphology. Representative data showed that double staining cells were regarded as debris (upper), while CD133 (+) cells were stained with AF488 only (lower).

**Figure 4.**
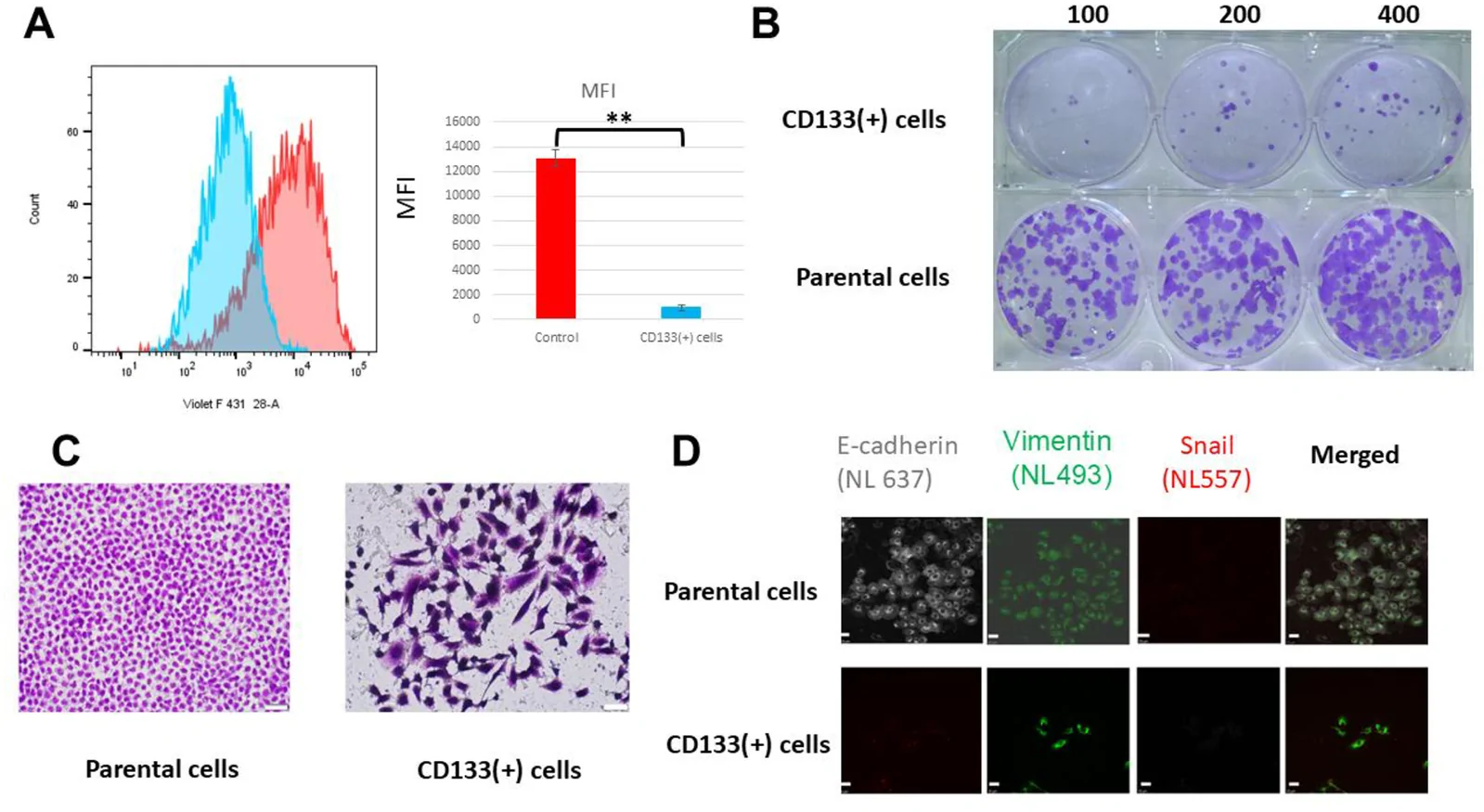
The sorted CD133(+) DU145 cells have a distinct phenotype compared to control cells *in vitro*. **(A)** The EpCAM expression of sorted CD133(+) DU145 cells compared to control cells. The data was presented in FC histogram (left) and MFI value (right). Orange and blue lines in the flow cytometry histogram represent parental and CD133(+) cells stained with EpCAM antibody. Data are expressed as the mean ± standard deviation from 4 independent experiments. *p<0.05; **p<0.01. **(B)** The colonies of 100, 200, 400 cells formed from CD133(+) cells and control DU145 cells in a 2-week CFA assay. **(C)**The comparison of the morphology under microscopy between CD133(+) cells and control cells. Scale bar represents 50 μm. **(D)** Immunocytochemistry with an epithelial biomarker (E-cadherin) and two mesenchymal biomarkers (vimentin and snail), showing the reduced E-caderin expression and increased vimentin expression. Scale bar represents 25 μm.

**Figure 5.**
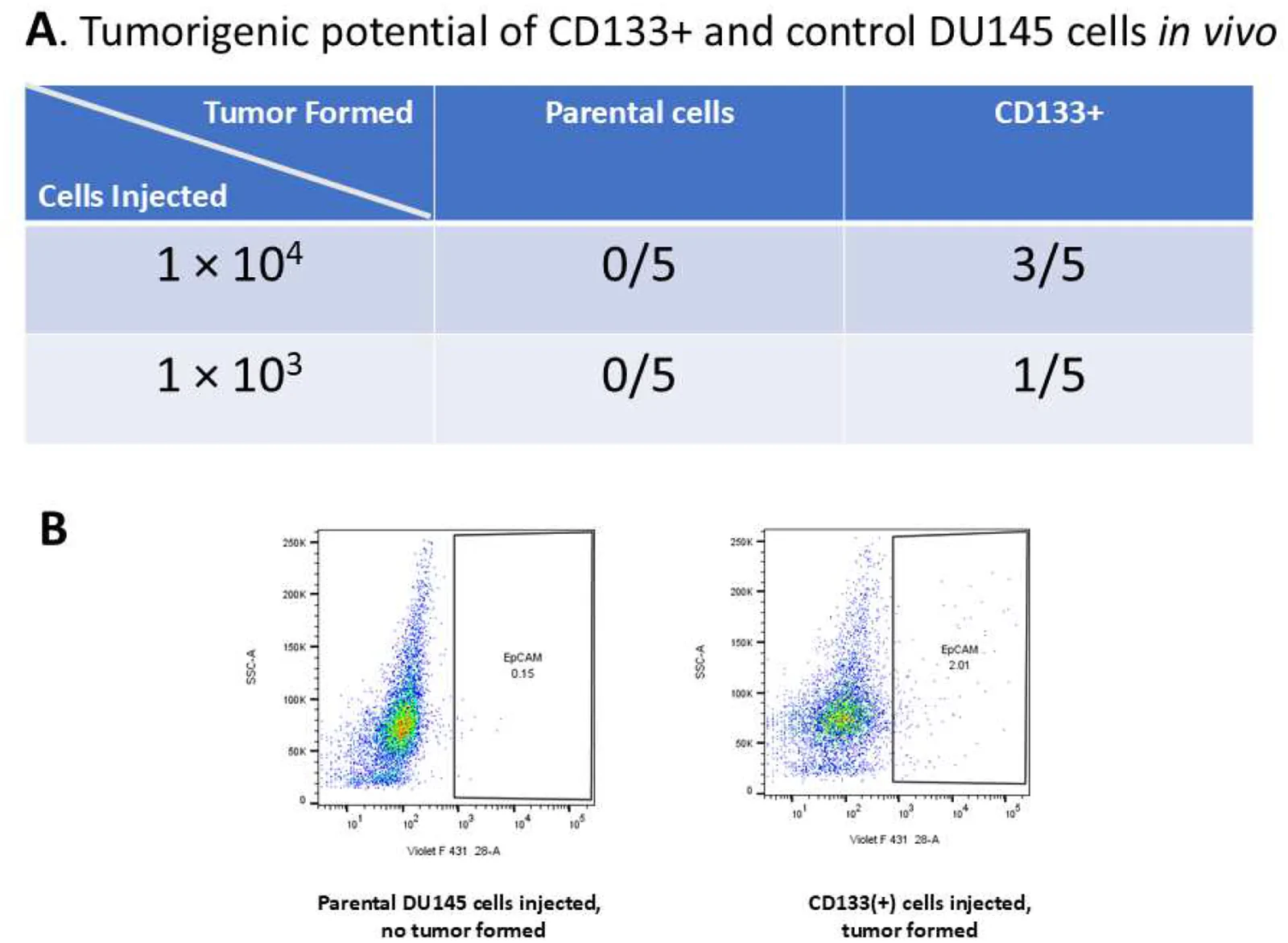
DWJM-induced CD133+ cells possess higher tumor-initiating capacity *in vivo*. **(A)** Cells were assayed for the ability to form tumors after subcutaneous injection into nude mice at 1 × 10^3^ or 1 × 10^4^ cells per injection. The number of tumors formed are indicated for each condition. ELDA software revealed that the tumor formation rate of CD133(+) cells was higher than that of parental DU145 cells (p < 0.01). **(B)** Elevated EpCAM expression was detected in 2/4 mice BM bearing tumor after inoculation with CD133(+) cells.

## Data Availability

The data that support the findings of this study is provided within the manuscript and raw data is available on reasonable request from the corresponding author.
